# Chronic Exposure to Low Frequency Noise at Moderate Levels Causes Impaired Balance in Mice

**DOI:** 10.1371/journal.pone.0039807

**Published:** 2012-06-29

**Authors:** Haruka Tamura, Nobutaka Ohgami, Ichiro Yajima, Machiko Iida, Kyoko Ohgami, Noriko Fujii, Hiroyuki Itabe, Tastuya Kusudo, Hitoshi Yamashita, Masashi Kato

**Affiliations:** 1 Unit of Environmental Health Sciences, Department of Biomedical Sciences, College of Life and Health Sciences, Chubu University, Kasugai, Aichi, Japan; 2 Research Reactor Institute, Kyoto University, Kumatori-cho, Sennan, Osaka, Japan; 3 Department of Biological Chemistry, Showa University School of Pharmacy, Hatanodai, Shinagawa-ku, Tokyo, Japan; 4 Unit of Molecular Biology, Department of Biomedical Sciences, College of Life and Health Sciences, Chubu University, Kasugai, Aichi, Japan; Université Pierre et Marie Curie, France

## Abstract

We are routinely exposed to low frequency noise (LFN; below 0.5 kHz) at moderate levels of 60–70 dB sound pressure level (SPL) generated from various sources in occupational and daily environments. LFN has been reported to affect balance in humans. However, there is limited information about the influence of chronic exposure to LFN at moderate levels for balance. In this study, we investigated whether chronic exposure to LFN at a moderate level of 70 dB SPL affects the vestibule, which is one of the organs responsible for balance in mice. Wild-type ICR mice were exposed for 1 month to LFN (0.1 kHz) and high frequency noise (HFN; 16 kHz) at 70 dB SPL at a distance of approximately 10–20 cm. Behavior analyses including rotarod, beam-crossing and footprint analyses showed impairments of balance in LFN-exposed mice but not in non-exposed mice or HFN-exposed mice. Immunohistochemical analysis showed a decreased number of vestibular hair cells and increased levels of oxidative stress in LFN-exposed mice compared to those in non-exposed mice. Our results suggest that chronic exposure to LFN at moderate levels causes impaired balance involving morphological impairments of the vestibule with enhanced levels of oxidative stress. Thus, the results of this study indicate the importance of considering the risk of chronic exposure to LFN at a moderate level for imbalance.

## Introduction

Exposure to noise generated in occupational and daily environments is one of the community hazards in our society [1], [2]. Noise consists of sound with broad frequencies, but there is limited information about the frequency-dependent influence of noise on health. Low frequency noise (LFN) is constantly generated from natural and artificial sources. The frequency range of LFN is usually defined as being below 100 Hz, while that of infrasound is usually below 20 Hz [3]. LFN is ubiquitously detected in our modern society and is generated from many occupational and daily sources including transportation systems, industrial devices, air movement devices (e.g., wind turbines, compressors, ventilation and air-conditioning units) and household appliances (e.g., washing machines, refrigerators and freezers). Thus, we are routinely exposed to LFN generated from various devices in the daily environment. In fact, our measurements showed LFN at moderate levels of about 70 dB sound pressure level (SPL) generated from various ordinary devices (Table 1).

Previous studies have indicated that LFN at below 0.5 kHz can be an environmental factor threatening health [4]. In humans, effects of LFN on several physiological functions including the cardiovascular and nervous systems, visual system, auditory system and the endocrine system have been shown [3]. Effects of LFN on the central nervous system including annoyance, sleep and wakefulness, perception, evoked potentials, electroencephalographic changes and cognition have also been shown [5], [6]. Chronic exposure to environmental infrasound has been shown to affect blood pressure, resulting in hypertension in humans [7]. Furthermore, exposure to moderate levels of LFN (70 dB SPL) at the frequency region of 31.5 Hz to 125 Hz for 2 hours has been shown to affect neuroendocrine activity in humans [8]. On the other hand, audible frequencies for humans and mice are known to be approximately 0.02–20 kHz and 1–40 kHz, respectively [9]. Therefore, it is basically difficult for people and mice even without hearing loss to recognize LFN in a noisy environment [3]. Thus, it is important to further analyze the potential risk of occupational and daily exposure to LFN at moderate levels on our health, even if we hardly recognize LFN in daily or occupational environments.

Balance is coordinately regulated by several organs including the vestibule, skeletal muscle and cerebellum [10]. A previous study showed that aging, injuries and other genetic factors can cause abnormal physiological functions in these crucial organs that result in impairments of balance in mice and humans [11], which have a negative impact on quality of life in an aging society. On the other hand, exposure to infrasound (5 Hz and 16 Hz, 95 dB, 5 minutes) has been shown to affect the control of upright standing posture in humans [12]. Also, occupational exposure to LFN has been shown to lead to impairments of vestibular functions [13]. Thus, these previous studies suggest that exposure to LFN can affect balance regulated by vestibular functions in humans. In previous studies with experimental animals, behavior analyses including rotarod, beam-crossing and footprint tests have been used to determine balance [14]–[16]. However, there is very limited information about how chronic exposure to LFN affects balance in mice.

**Table 1:** Typical low frequency noise levels of electric devices in experimental rooms.

Electric devices	Noise levels (dB SPL) at 100 Hz
4°C freezers	65.3±4.8
−30°C freezers	66.1±4.5
−80°C freezers	69.9±2.9
Ice makers	61.8±1.0
Draft chambers	75.5±0.7

Noise levels (means ± SD) were measured by a noise level meter and calculated as an average of five repeated measurements. Noise levels were measured at a distance of approximately 20 cm from the devices shown in Table 1. Background level (mean ± SD) of low frequency noise at 100 Hz was 35.7±2.7 dB SPL in 5 experimental rooms without noise-generating devices shown in Table 1.

Inner ears contain the vestibule in the vicinity of the organ of Corti. Vestibular hair cells covered with otoconia play an important role in mechanotransduction, by which gravity impulses are converted into neural impulses. Impairments of vestibular hair cells have been shown to cause abnormal behaviors including balance [16]. Thus, the vestibule containing hair cells and an otolith is one of the organs responsible for balance. On the other hand, exposure to a broadband noise (at 1–20 kHz) has been shown to induce ototoxic damage of hair cells with enhanced oxidative stress in the organ of Corti in the inner ear, resulting in noise-induced hearing loss in rodent animal models and humans [17]. In addition, exposure to broadband noise has been shown to enhance oxidative stress in the brain [18], [19]. Thus, it is possible that exposure to noise causes damage of hair cells with increased oxidative stress in inner ears, although those previous studies used broadband noise without consideration of specific frequencies. At present, however, there is no information about whether exposure to LFN enhances oxidative stress in vestibular hair cells, which play a crucial role in regulation of balance.

**Figure 1:**
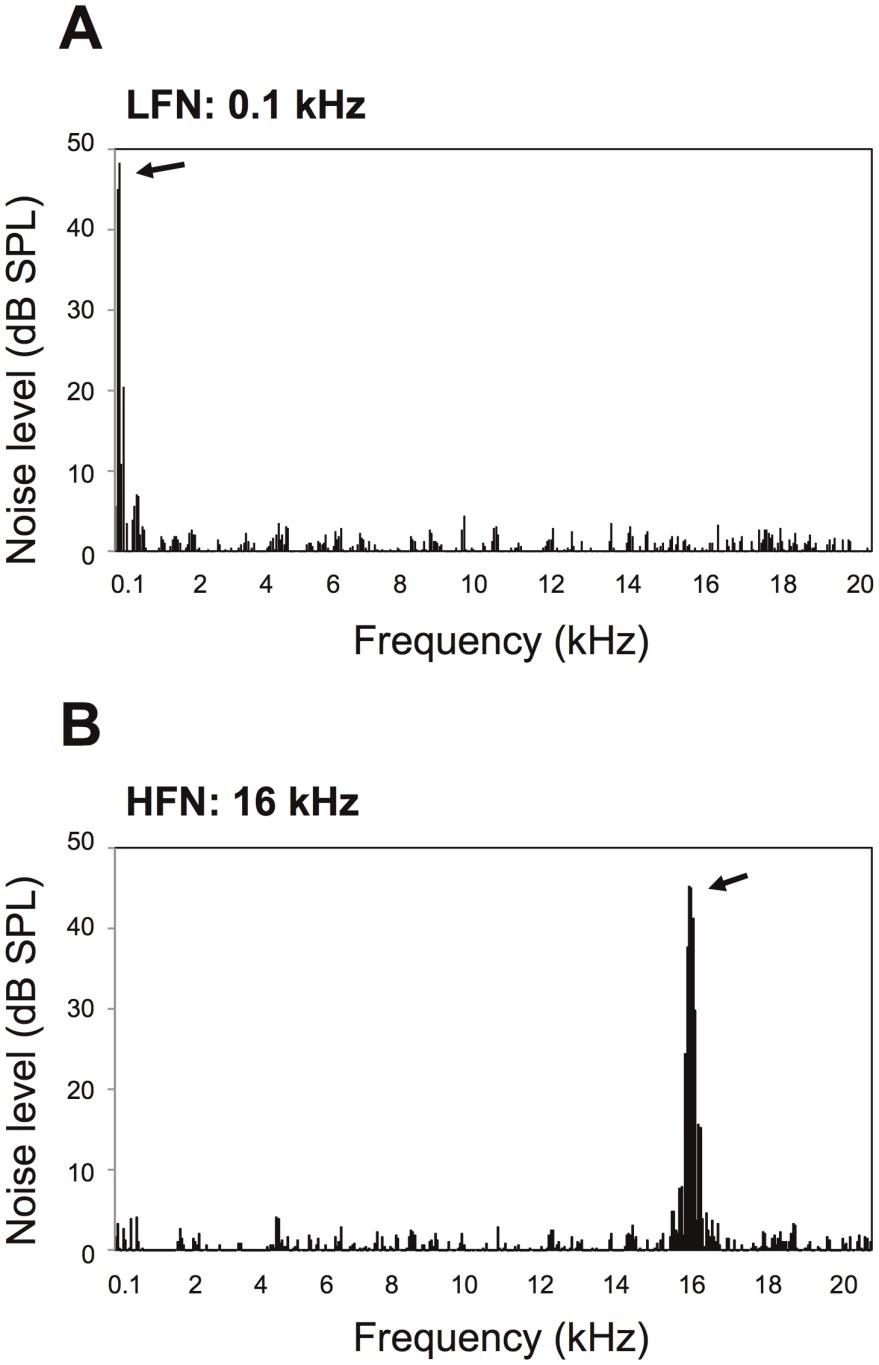
Frequency distributions of noise used in this study. Frequency distributions (means ± SD) of (A) low frequency noise (LFN; 0.1 kHz) and (B) high frequency noise (HFN; 16 kHz) are presented. Noise levels from a speaker at a distance of 10 cm in a soundproof room were measured by a noise level meter and calculated as average of five repeated measurements. Background levels measured in a soundproof room without noise-generating devices were subtracted from noise levels from the speaker. Whole noise levels of (A) LFN and (B) HFN measured by the noise level meter without FFT analyzing software were almost the same (70 dB SPL).

In this study, we used LFN (0.1 kHz) and high frequency noise (HFN; 16 kHz) at a moderate level of 70 dB SPL [20] for exposure of mice to noise (Fig. 1) in order to determine the pathogenesis of impaired balance caused by LFN stress. Our results show for the first time that chronic exposure to LFN at a moderate level can cause impaired balance involving partial loss of hair cells with increased levels of oxidative stress in the vestibule.

## Results

### Chronic Exposure to LFN at a Moderate Level Affects Balance in Mice

**Figure 2:**
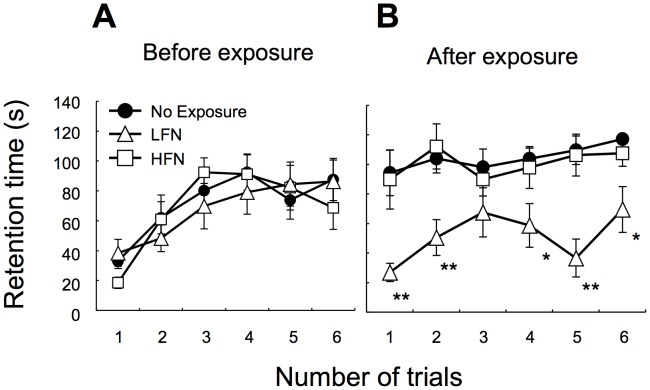
Exposure to LFN affects rotarod performance of ICR mice. Before (A) and after (B) exposure to LFN (open triangles) and HFN (open squares), retention times (seconds, mean ± SD, n = 7) on the rotarod (at 30 rpm) were measured. Results for non-exposed mice (closed circles) are also plotted (mean ± SD, n = 7). Mice were allowed a maximum retention time of 120 seconds per trial. Significant difference (**, *p*<0.01; *, *p*<0.05) from the non-exposure group was analyzed by the Mann-Whitney *U* test.

**Figure 3:**
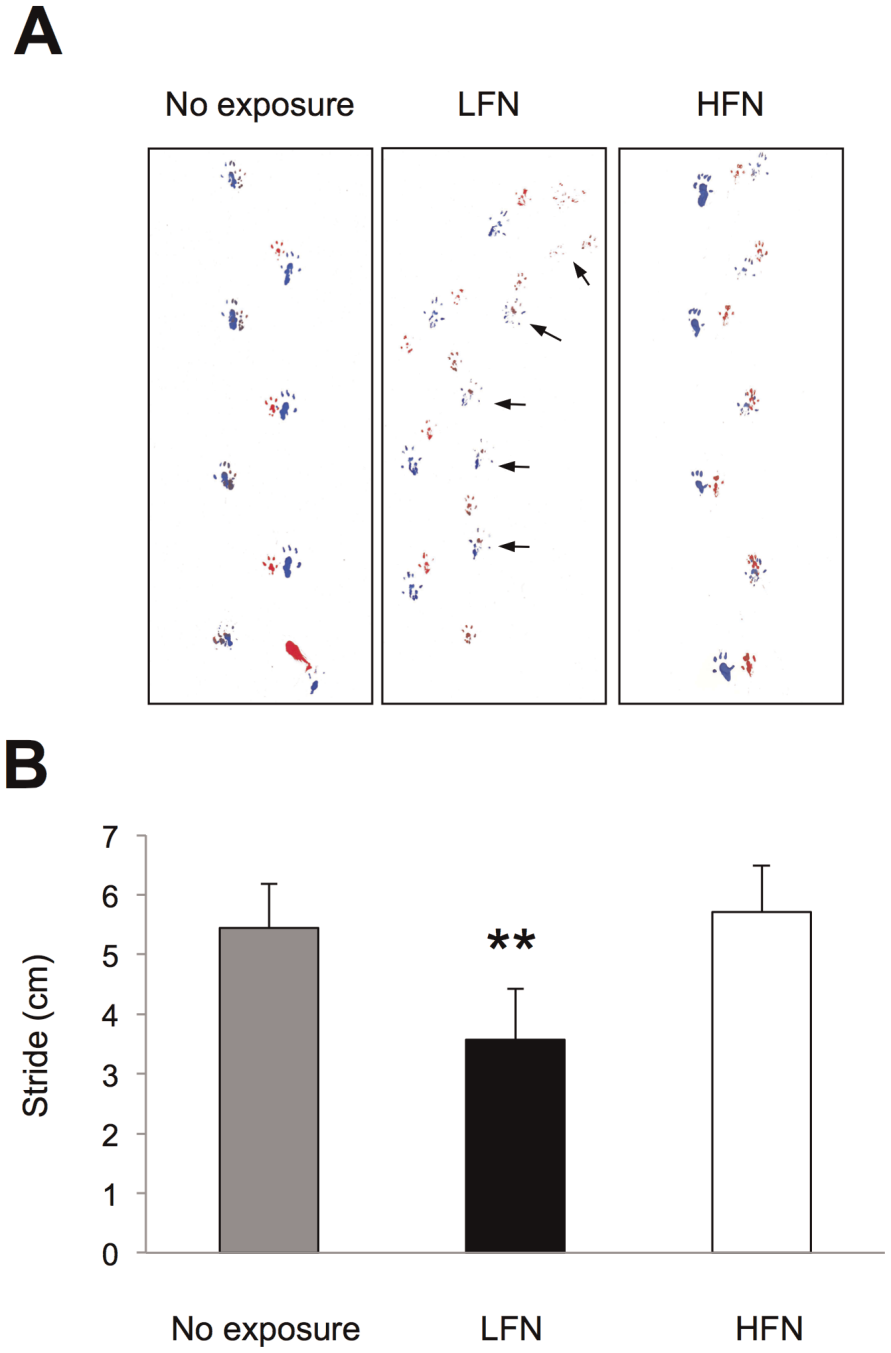
Exposure to LFN affects gait pattern of ICR mice. (A) After exposure to LFN (center panel) and HFN (right panel), front and back paws of mice were dipped in red or green paint, and mice walked across a box lined with paper. Non-exposed mice (left panel) are also shown. LFN-exposed mice display shorter stride length and winding gait patterns (center panel, arrows). (B) Quantification of stride length. Strides (mean ± SD) for seven mice (each group) were assessed. A total of 40–50 steps for each group were determined. Significant difference (**, *p*<0.01) from the non-exposure group was analyzed by the Mann-Whitney *U* test.

We started to expose ICR mice to 70 dB SPL of LFN (0.1 kHz, Fig. 1B) and HFN (16 kHz, Fig. 1A) from 6 weeks of age in order to determine how exposure to noise affects balance in a frequency-dependent manner. After noise exposure, we performed behavior analyses to determine whether exposure to LFN affects balance in mice. Comparable rotarod performance was observed in non-exposed and exposed mice before noise exposure (Fig. 2A). LFN-exposed mice showed significantly worse rotarod performance than that of non-exposed mice (Fig. 2B). In contrast, mice exposed to HFN and non-exposed mice showed comparable rotarod performances (Fig. 2B). The beam-crossing test also showed imbalance behaviors in LFN-exposed mice compared to those in non-exposed mice and HFN-exposed mice (Table 2). Footprint analysis further showed winding gait patterns and short strides in LFN-exposed mice (Fig. 3A, center panel) compared to those in non-exposed mice and HFN-exposed mice (Fig. 3B). These results suggest that LFN-exposed mice had impaired balance. An abrupt change of body weight was not observed during noise exposure (Fig. S1).

**Table 2:** Beam crossing test for LFN-exposed mice.

	No exposure	HFN	LFN
Success	12	10	2
Failure	0	0	10**

ICR mice that had been exposed to LFN (n = 12) and to HFN (n = 10) and non-exposed mice (n = 12) were examined by a beam crossing test. The number of mice that fell from the beam (i.e., imbalance behavior) is shown as “Failure” in Table 2. The number of mice crossing the beam without falling is shown as “Success” in Table 2. P values were obtained by chi square analysis. **p<0.01.

### Chronic Exposure to LFN at a Moderate Level Causes Partial Loss of Vestibular Hair Cells

**Figure 4:**
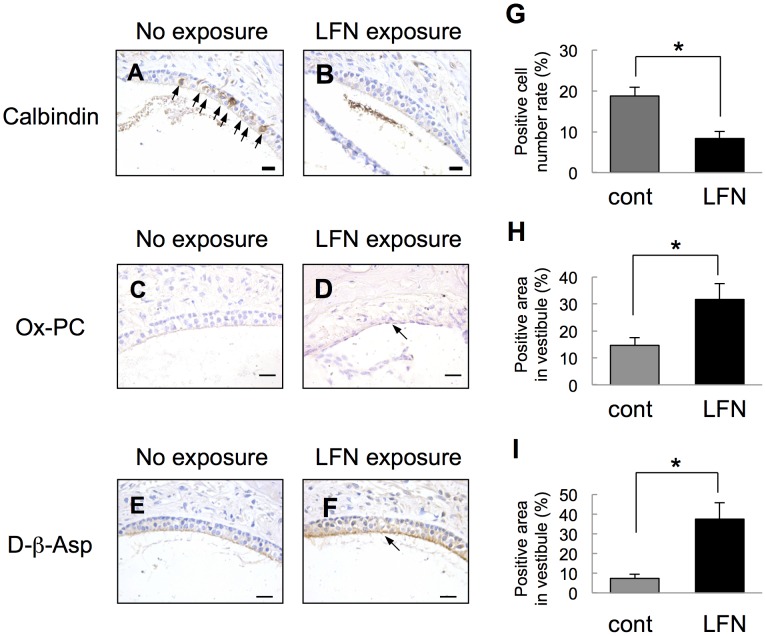
Decreased number of vestibular hair cells with increased levels of oxidative stress in LFN-exposed mice. (A, B) Immunohistochemical analysis with anti-calbindin D28k for vestibules in LFN-exposed (B) and non-exposed mice (A). (C-F) Enhanced oxidative stress levels in vestibules of LFN-exposed mice. Vestibules of LFN-exposed (D, F) and non-exposed mice (C, E) were immunohistochemically stained by an anti-Ox-PC antibody (DLH3) (C, D) and anti-D-βeta-Asp antibody (E, F). Vestibules of LFN-exposed mice showed stronger signals (D, F, arrows) than those of non-exposed mice (C, E). Scale bars: 20 µm. (G-I) Percentage (means ± SD) of calbindin-positive hair cells (G) and positive areas of anti-Ox-PC antibody (H) and anti-D-βeta-Asp antibody (I) in vestibules from LFN-exposed mice (LFN, black bar, n = 7) and non-exposed mice (Cont, gray bar, n = 7). Significant difference (*, *p*<0.05) from non-exposed mice was analyzed by the Mann-Whitney *U* test.

We then performed morphological analyses of the vestibule in inner ears from ICR mice after LFN exposure (Fig. 4). Immunohistochemical analyses with anti-calbindin D28k antibody, a marker for vestibular hair cells [21], showed a significant decrease in the number of vestibular hair cells in LFN-exposed mice (Fig. 4B) compared to that in non-exposed mice (Fig. 4A, arrows, 4G). Furthermore, we histologically determined oxidative stress levels of the vestibule in LFN-exposed mice. Immunohistochemistry with anti-oxidized phospholipid (Ox-PC) antibody [22], [23] and anti-D-beta-aspartic acid (D-βeta-Asp) antibody [24] showed much stronger signals in marginal zones of the vestibule in LFN-exposed mice than in non-exposed mice (Fig. 4C-F, H, I).

## Discussion

In previous studies, exposure to LFN has been shown to lead to impairments of balance in humans [4], [12]. However, there is very limited information about the etiology of imbalance caused by LFN and the influence of a moderate level of LFN on balance since it is basically difficult to reveal the pathogenesis of impaired balance caused by LFN stress in humans. This study showed for the first time that chronic exposure to LFN at a moderate level causes imbalance involving morphological impairments in the vestibule in mice.

This study showed that exposure to LFN caused impairments of balance in mice, whereas mice exposed to HFN for 1 month and non-exposed mice showed comparable performances related to balance. A previous study showed that energy of LFN (∼ 0.5 kHz) can penetrate our body, whereas HFN is easily attenuated [25]. Therefore, we assume that LFN stress causes damage to the vestibule due to its higher energy than that of HFN. On the other hand, there has been no study showing the influence of noise on behavior and morphology of the responsible tissues in mice or humans by consideration of both frequency (Hz) and intensity (dB). A previous study in which the influence of LFN on sleep in humans was investigated showed that 1 kHz noise only at 30 dB increased wakefulness, while 42 Hz LFN even at 70 dB had little effect [5]. Since audible frequencies for humans are known to be approximately 0.02–20 kHz, it is basically harder for people even without hearing loss to recognize noise at 42 Hz than that at 1 kHz. Thus, it is possible that not only frequency and intensity of noise but also the range of audible frequencies of the recipient affect physiological functions including balance. Therefore, it is also important to measure changes in auditory responses to noise by auditory brain stem responses (ABR) in frequency- and intensity-dependent manners. Further study is needed to determine the influence of noise on balance and morphology of the responsible tissues in mice and humans by consideration of frequency (Hz), intensity (dB) and the range of audible frequencies of mice and humans.

In a previous study, several organs including muscles, the cerebellum and the vestibule were shown to coordinately regulate balance [10]. Our results obtained with light microscopy showed comparable morphologies of soleus muscle fibers and the cerebellum in exposed and non-exposed mice (Fig. S2, Method S1). Body weights of LFN-exposed and non-exposed mice were not different (Fig. S1). Thus, our results suggest that LFN causes morphological impairment of the vestibule rather than the soleus muscle and cerebellum. In previous studies, exposure to LFN has been suggested to ubiquitously affect several organs in our body [3]. Also, exposure to a moderate level of LFN (70 dB SPL) for 2 hours has been shown to influence neuroendocrine activity related to emotional stress in humans [8]. Therefore, it would be interesting to determine whether chronic exposure to LFN stress affects psychological functions relevant to balance. One the other hand, female mice were used for exposure experiments in this study. It has been reported that hormonal changes during the menstrual cycle in females affect behaviors including depression symptoms in humans and mice [26], [27]. As far as we measured behavior in female and male mice, the two sexes showed similar susceptibility to the influence of LFN exposure on balance (data not shown). Our results partially correspond to the results of a previous study showing that performance of a visual inspection task was not affected by any combinational effect or interaction between menstrual cycle and exposure to broadband noise in humans [28]. It would be of interest to further investigate whether chronic exposure to LFN stress affects hormonal functions including physiological functions relevant to balance.

The inner ears contain the organ of Corti in the vicinity of the vestibule. The organ of Corti, which consists of two kinds of sensory cells [inner hair cells (IHCs) and outer hair cells (OHCs)], is a sound receptor by which sound impulses are converted into neural impulses. Auditory information from sensory cells is eventually transmitted to the auditory cortex [29], where auditory stimuli activate other parts of the cortex including the amygdala and thalamus. Thus, exposure to LFN can simultaneously stimulate not only the vestibule, directly causing impairment of balance, but also the organ of Corti, affecting behavior indirectly via the central nervous system through the auditory system. Additional experiments are required to determine whether exposure to LFN affects vestibular functions separately from the auditory system.

In this study, we showed that impairment of balance involved partial loss of calbindin-positive hair cells in the vestibule with enhanced oxidative stress in LFN-exposed mice. Our results partially correspond to results of previous studies showing that behavioral impairments induced by antibiotics were accompanied by degeneration of vestibular cells and oxidative stress [30], [31]. Also, a previous study has shown that antioxidant compounds have preventive effects on noise-induced hearing loss [17]. Ototoxicity caused by oxidative stress in inner ears usually has been shown to involve impairments of antioxidant enzymes [17]. Thus, those previous studies indicate the necessity for further investigation of a causal molecule related to oxidative stress in vestibular hair cells affected by LFN and a preventive effect of antioxidants on impaired balance caused by LFN exposure. On the other hand, a previous study showed that calbindin D28k serves as a calcium buffering protein to maintain a low endolymphatic calcium [Ca^2+^] level in inner ears [32]. In addition, a low luminal calcium concentration ([Ca^2+^]) as well as a high endolymphatic potassium concentration ([K^+^]) of the mammalian endolymph in the inner ear are required for normal balance [32], [33]. Endolymphatic levels of these ions are maintained by channels, transporters and buffering proteins expressed in inner ears [33]. Rats exposed to a toxic compound have been shown to have a decreased number of calbindin D28k-positive neurons with enhanced oxidative stress in the cerebellum [34]. Interestingly, infrasound has been shown to cause impairments of [Ca^2+^] levels in rat cardiac tissues resulting in cardiac dysfunction [35]. Based on our results and the results obtained in previous studies, we hypothesize that partial loss of calbindin-positive hair cells in the vestibule caused by LFN exposure may involve impairments of endolymphatic [Ca^2+^] levels resulting in imbalance.

Exposure of children to environmental factors has been shown in previous studies to sensitively affect auditory development [36], [37]. Aging has also been shown to change susceptibility to ototoxic factors in mice [38]. On the other hand, there is very limited information about the relevance between age and imbalance caused by exposure to LFN, while a previous study suggested that young children have a high susceptibility to LFN [39]. Therefore, our results indicate the necessity for further investigation of the age-specific susceptibility of imbalance to LFN in mice and humans.

In conclusion, our results demonstrate for the first time that chronic exposure of ICR mice to LFN at 70 dB SPL for 1 month results in a decreased number of vestibular hair cells with enhanced levels of oxidative stress, leading to in impairment of balance.

## Materials and Methods

### Mice

Randomly bred wild-type female mice (ICR) at 6 weeks of age were used for exposure experiments. Mice were purchased from Japan SLC (Hamamatsu, Japan). All experiments were authorized by the Institutional Animal Care and Use Committee in Chubu University (approval number: 2410030) and followed the Japanese Government Regulations for Animal Experiments.

### Noise Exposure

Mice were continuously exposed for 1 month to noise at 70 dB SPL from a speaker (Sound Stimulator DPS-725, Diya Medical System CO., LTD, Japan) with the mice being located at a distance of approximately 10–20 cm from the speaker in a soundproof room (Yamaha Co., Japan). The acoustic output was regularly monitored using a noise level meter (Type 6224 with an FFT analyzer, ACO CO., LTD, Japan). Mice were housed under specific pathogen-free (SPF) conditions at a constant temperature (23±2°C) and a 12-h light/dark cycle. The mice were weighed weekly on a gram scale.

### Behavior Analyses

Measurement of balance was performed according to previous studies [14]–[16]. Before and after noise exposure for 4 weeks, mice were examined using a rotating rod treadmill (Ugo Basile; Stoelting Co., Chicago, IL). Mice with similar body weights were used. The rotating rod was set in motion at a constant speed (30 rpm) and the mice were placed into individual sections of the rotating rod. Each time an animal fell, it was noted whether the fall had occurred when it sat still or when it walked. Each animal’s performance score in seconds was recorded when the mouse was unable to stay on the rotating rod, tripped a plate and stopped the timer. Six successive trials separated by 5-min pauses were performed. For the beam-crossing test, a round wooder bar of 2 cm in diameter was attached to two Styrofoam platforms at the ends, and the length of the bar to be crossed was adjustable at the ends. Pre-training of mice on the bar at 5 cm in length was performed first, followed by three consecutive trials of crossing the bar at 30 cm in length. Mice were allowed up to 60 sec to traverse each beam. A gait assay was performed as previously reported [14]. Briefly, the front paws were dipped in red paint and the back paws were dipped in blue paint, and mice were placed on Whatman paper at one end of a 14×44 cm box. The distance between the back edge of each same-side paw print was used to determine stride length.

### Morphological Analyses of the Vestibule

After perfusion fixation by Bouin’s solution, inner ears with semicircular canals from mice were immersed in the same solution for 3 days to 1 week at 4°C. Immunohistochemistry with rabbit anti-calbindin D28k antibody (1∶150; Chemicon) [40] and rabbit anti-D-beta-aspartic acid (D-βeta-Asp) antibody (1∶100) [24] was performed with 5-µm-thick serial paraffin sections. A VECTASTAIN ABC kit (Vector) and an Envision kit/HRP (diaminobenzidine; DAB) (DAKO) were used in each immunohistochemical analysis with hematoxylin counterstaining. Immunohistochemical analyses with a monoclonal antibody against oxidized phospholipids (Ox-PC; 20 µg/ml; DLH3) [22] were performed for frozen sections [23]. Briefly, after treatment with 0.3% hydrogen peroxiside for 20 min at room temperature, the frozen sections were incubated in blocking buffers (20 µg/ml of goat IgG (DAKO) in PBS for 1 h at room temp, followed by 20 µg/ml of Goat Fab-anti mouse IgG (Jackson Immunoresearch)). After incubation with the primary antibody, the frozen sections were further incubated in an alkaline phosphatase-labeled goat anti-mouse IgM (1∶200 in 2% skim milk in PBS; AbD serotec). The DAKO New Fuchsin substrate system (DAKO) was used to develop a signal with hematoxylin counterstaining. About 5 sections were observed in seven exposed and seven non-exposed mice, respectively. The percentage of positive signals histochemically detected by antibodies was estimated with WinROOF (version 6.2) as previously reported [40], [41]. Briefly, the number of positive hair cells was divided by the total number of hair cells in the vestibule. A total of 100–150 cells in 5 sections from each mouse were examined. In the case of oxidative stress markers including Ox-PC and D-βeta-Asp, positive areas in the measured vestibule were divided by the areas of the section measured. Vestibules from seven mice for each group were measured for each estimation.

### Statistics

Statistical analysis was performed as previously reported [42]. Significant difference (**, *p*<0.01; *, *p*<0.05) from the control (no exposure) was analyzed by the Mann-Whitney *U* test (Figs. 2, 3, and 4 and Fig. S1) and chi square analysis (Table 2).

## Supporting Information

Figure S1 **Exposure to low frequency noise does not affect body weight of ICR mice.** Body weights (mean ± SD) were monitored at 1 week (*1W*), 2 weeks (*2W*), and 4 weeks (*4W*) during exposure to LFN. No significant difference (n.s.) of body weight was observed in LFN-exposed and non-exposed mice.
(TIFF)

Figure S2 **Morphological analysis of cerebellum and soleus muscle in LFN-exposed and non-exposed mice.** (A, B) Hematoxylin-eosin (HE) staining of the cerebellum in LFN-exposed (B) and non-exposed mice (A) was performed with 5-µm-thick serial paraffin sections. (C, D) NADH-TR staining for the soleus muscle in exposed (D) and non-exposed mice (C) was performed. Scale bars: 20 µm (A, B), 50 µm (C, D).
(TIFF)

Methods S1 **NADH-tetrazolium (NADH-TR) staining.**
(DOC)
