# Supplementary figures and images for: Chronic Exposure to Low Frequency Noise at Moderate Levels Causes Impaired Balance in Mice
Source: PLoS One. 2012 Jun 29;7(6):e39807. doi: 10.1371/journal.pone.0039807 (PMC3387207; doi:10.1371/journal.pone.0039807)

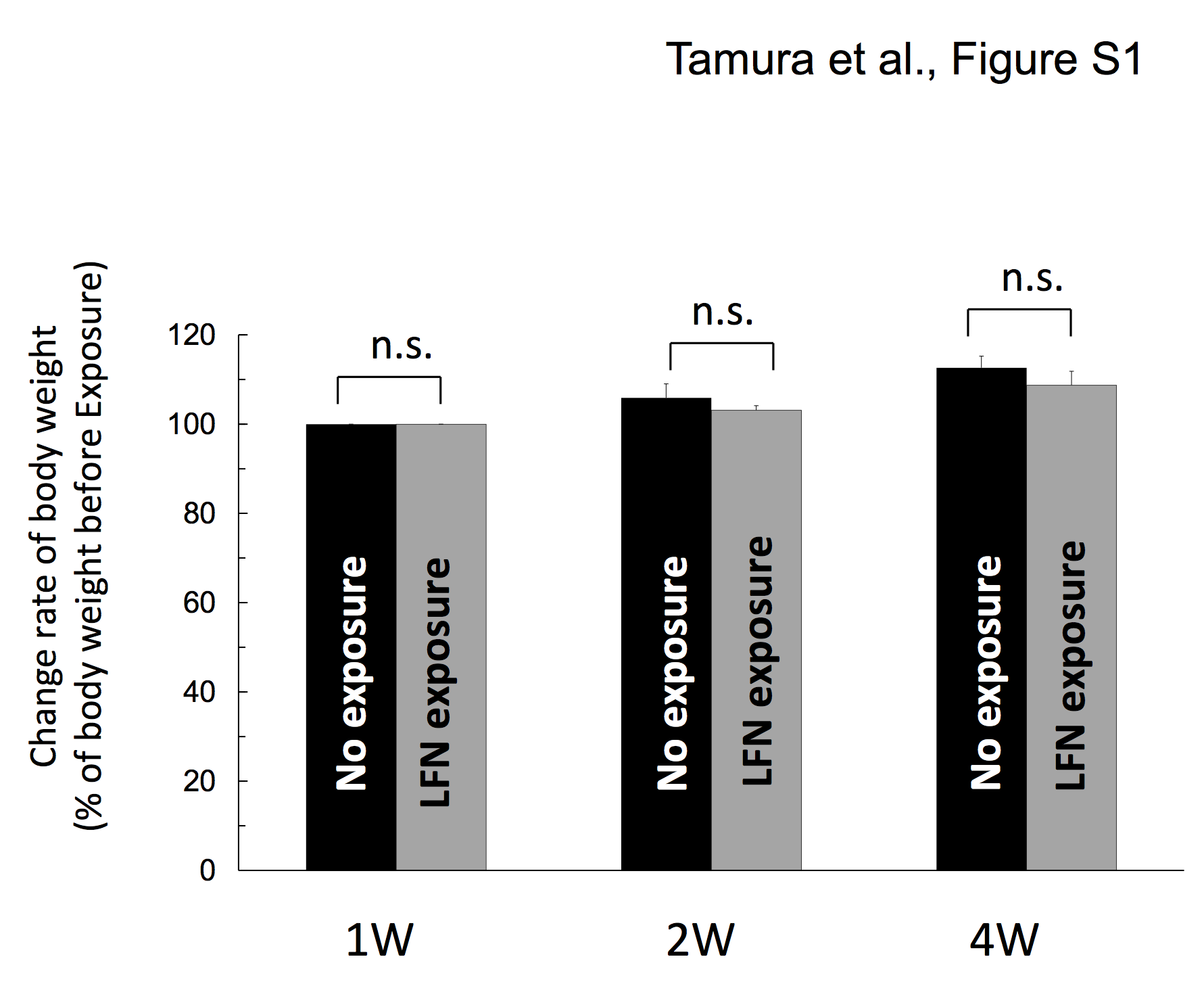

Supplement: Figure S1 — Exposure to low frequency noise does not affect body weight of ICR mice. Body weights (mean ± SD) were monitored at 1 week (1W), 2 weeks (2W), and 4 weeks (4W) during exposure to LFN. No significant difference (n.s.) of body weight was observed in LFN-exposed and non-exposed mice. (TIFF) [file pone.0039807.s001.tiff]

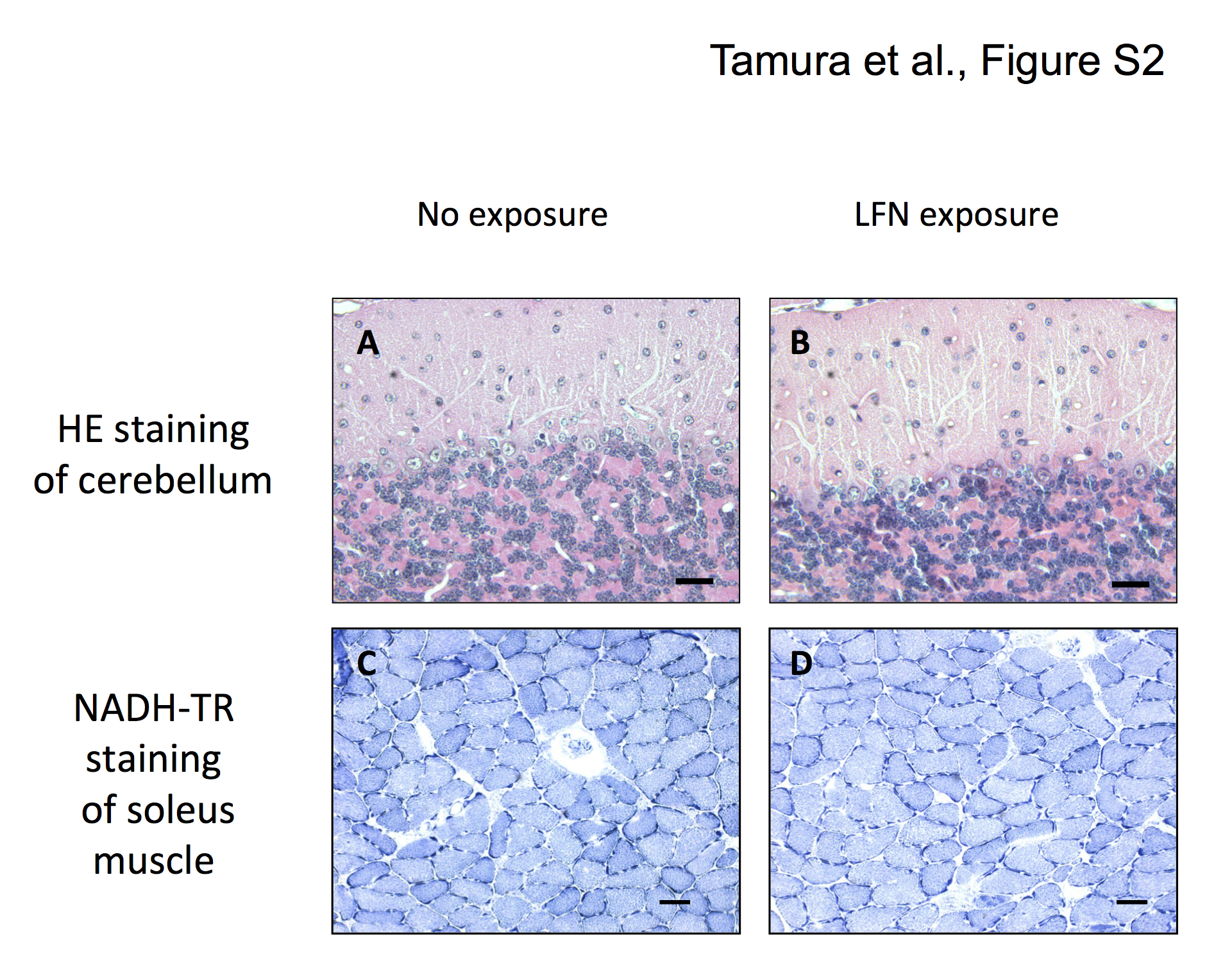

Supplement: Figure S2 — Morphological analysis of cerebellum and soleus muscle in LFN-exposed and non-exposed mice. (A, B) Hematoxylin-eosin (HE) staining of the cerebellum in LFN-exposed (B) and non-exposed mice (A) was performed with 5-µm-thick serial paraffin sections. (C, D) NADH-TR staining for the soleus muscle in exposed (D) and non-exposed mice (C) was performed. Scale bars: 20 µm (A, B), 50 µm (C, D). (TIFF) [file pone.0039807.s002.tiff]
