# Supplementary material for: Chronic Exposure to Low Frequency Noise at Moderate Levels Causes Impaired Balance in Mice
Source: PLoS One. 2012 Jun 29;7(6):e39807. doi: 10.1371/journal.pone.0039807 (PMC3387207; doi:10.1371/journal.pone.0039807)
Supplement: Methods S1 — NADH-tetrazolium (NADH-TR) staining. (DOC) [file pone.0039807.s003.doc]

**Methods S1. NADH-tetrazolium (NADH-TR) staining.** Mice were sacrificed by cervical dislocation. Soleus muscles were immediately immersed in isopentane under liquid nitrogen. After embedding with OCT compound (Sakura Finetechnical, Japan), serial cryosections (6 µm in thickness) were prepared. Tissue sections were incubated for 30 min at 37°C in a solution of 0.06 M Tris-HCl (pH 7.4), 0.5 mM beta-NADH (Sigma), and 0.6 mM nitro blue tetrazolium (WAKO). Sections were then serially incubated with acetone solutions (30, 60, 90, 60, and 30% for 1 min each). Five hundred to 1,000 fibers from approximately five random fields within the tissue section were observed.
